# Monoclonal antibody desensitization in a patient with a generalized urticarial reaction following denosumab administration

**DOI:** 10.1186/s13223-015-0097-6

**Published:** 2015-10-26

**Authors:** D. Gutiérrez-Fernández, María-Jesús Cruz, A. Foncubierta-Fernández, A. Moreno-Ancillo, M. J. Fernández-Anguita, F. Medina-Varo, J. A. Andres-García

**Affiliations:** Pneumology-Allergy Department, Puerta del Mar University Hospital, Cádiz, Spain; Servei de Pneumologia, Hospital Universitari Vall d’Hebron, Passeig Vall d’Hebron, 119, 08035 Barcelona, Spain; Joaquín Pece Primary Care Centre, San Fernando, Cádiz Spain; Allergy Department, Hospital Nuestra Señora del Prado, Talavera de la Reina, Toledo Spain; Hospital Pharmacy, Puerta del Mar University Hospital, Cádiz, Spain; Unidad de Gestión Clínica Aparato Locomotor, Rheumatology Department, Puerta del Mar University Hospital, Cádiz, Spain; Unidad de Gestión Clínica Aparato Locomotor, Orthopaedic Surgery and Traumatology Department, Puerta del Mar University Hospital, Cádiz, Spain

**Keywords:** mABs, Hypersensitivity reaction, Osteoporosis

## Abstract

Denosumab is a human monoclonal antibody indicated for the treatment of osteoporosis in postmenopausal women with a high risk of fractures. To our knowledge, no cases of desensitization to this drug have been described in the literature. We report the first case of generalized urticarial reaction and facial angioedema after therapy with denosumab. A subcutaneous desensitization protocol was successfully completed in this patient. Rapid desensitization is a promising method for the delivery of denosumab after a hypersensitivity reaction, and should be considered in osteoporosis treatment when no acceptable therapeutic alternatives are available.

## Background

Denosumab is a monoclonal antibody (mAb) used in the treatment of osteoporosis, approved by the FDA and the European Medicines Agency in 2010 [1]. The infusion of this mAb may cause urinary tract infections and upper respiratory tract infections, sciatica, cataracts, constipation, limb pain and skin rashes, including a high incidence of eczema (3 %) [2]. The use of monoclonal antibodies is increasing but, despite their clinical utility, they have been associated with hypersensitivity reactions [3]. We present a desensitization protocol for denosumab in a patient with a generalized urticarial reaction and facial angioedema following the administration of this drug. To our knowledge, no cases of desensitization to denosumab have been described to date.

## Case

**Fig. 1:**
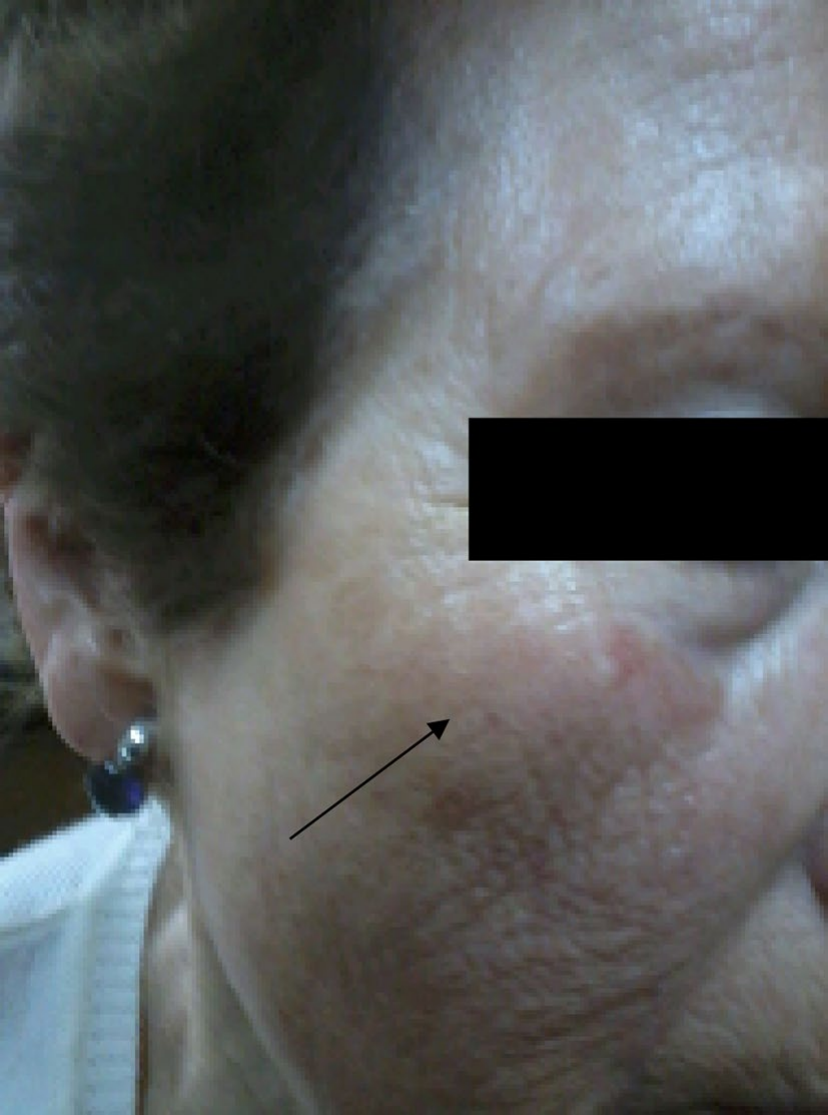
Bilateral facial angioedema one day after the first administration of denosumab

We report the case of a 65-year-old woman with a history of hysterectomy at age 35, who presented generalized osteoporosis, severe bone pain and risk of bone fractures. The patient was referred from trauma management after failure of prior treatments for osteoporosis (bisphosphonates, alendronate and risedronic acid) after major side effects: gastrointestinal erosion with biphosphonates and alendronate, and severe gastrointestinal bleeding, severe muscle and joint pain, fever, flu symptoms, conjunctivitis, episcleritis and uveitis with risedronic acid. All symptoms were intense and persistent. On the day after the first administration of denosumab the patient developed a generalized urticarial rash (thighs, abdomen, bilateral breast area, back) accompanied by bilateral facial angioedema and pruriginous injuries in the area of drug administration (the abdomen) (Fig. 1). The symptoms started 2 h after the first administration and resolved completely after 15 days with the administration of antihistamines and oral corticoids and the application of local corticoids.

Informed consent was obtained from the patient to perform an allergy test. Skin prick test (SPT) with denosumab was performed 8 weeks after the initial adverse reaction in order to minimize the risk of false negative results. A commercial preparation of Prolia (Amgen Europe B.V., Breda, Netherlands; GlaxoSmithKline) was used, containing 60 mg of denosumab in each preloaded 1 ml syringe (60 mg/ml). Histamine prick (10 mg/ml) and saline solution were used as positive and negative controls. A positive reaction was defined as a wheal with a diameter at least 3 mm larger than that obtained by a negative control. For SPT, a drop of denosumab (60 mg/ml) was applied and pricked to the volar surface of the forearm, eliciting a negative response. For intradermal testing, denosumab was diluted in normal saline. We proceeded with intradermal injections of 0.03 ml of 1/100 and 1:10 dilution of the denosumab strength solution, which obtained negative results. These concentrations proved to be non-irritating in ten control subjects who had never been treated with denosumab. The patch test with undiluted drug was applied at both the upper back and the previous local injection site, and also produced negative results. Skin prick test to house dust mites, cat and dog dander, olive, grass pollen and latex were negative, as was serum specific IgE to latex dander.

Given the risk of bone fracture and the numerous side effects of the conventional therapies for the treatment of osteoporosis, the patient agreed to denosumab treatment using a desensitization protocol. After informed consent was given, the desensitization procedure was performed. Blood pressure and heart rate were monitored during the test. The patient was pre-medicated with methylprednisolone (40 mg IV), ranitidine (50 mg IV) and dextrochloropheniramine (5 mg IV) 1 h before drug administration. A desensitization protocol was begun with an initial subcutaneous dose of 0.005 mg which was gradually increased in an eight-step cycle until a cumulative dose of 60 mg was reached (Table 1). The interval between doses was 15 min and the process was completed in 2 h. No local or systemic reactions were observed, either immediate or delayed. Since then, the patient has tolerated other two cycles of denosumab desensitization using only antihistamines for premedication, and her osteoporosis has evolved favourably.

**Table 1:** Desensitization protocol for denosumab

Dose (mg)	Cumulative dose (mg)	Time (min)	Blood pressure (mmHg)	Arterial pulse (mg)
0.005	–	0	166/77	87
0.05	–	15	152/65	84
0.5	–	30	147/73	80
1.5	1.5	45	145/73	67
3	4.5	60	150/72	73
7.5	12	75	166/82	70
15	27	90	149/81	68
33	60	105	144/73	76

No adverse reactions were observed during the desensitization process

Blood pressure at baseline: 149/77; arterial pulse at baseline: 89

The four higher doses were prepared introducing the necessary volume from the original syringe (60 mg/ml) into four insulin syringes (0.55, 0.25, 0.125 and 0.05 ml respectively). For the four smaller doses, appropriate dilutions were made with saline solution, starting from the original syringe. The solutions were valid for 24 h, and were protected from light and refrigerated

In order to demonstrate the efficacy of our desensitization procedure, a denosumab provocation was performed 6 months after the third desensitization protocol. After 3 h the patient presented bilateral angioedema, which had not occurred in the previous cycles of denosumab desensitization. On the next day, the patient had a very pronounced angioedema of the eyelids and cheekbones. Tryptase levels were 2.3 ng/ml (negative up to 15 ng/ml) before the challenge test, 2.1 ng/ml after the challenge test, and finally 2.2 ng/ml after desensitization. Applying our desensitization procedure once again using only antihistamines as premedication, the drug was perfectly tolerated.

## Discussion

We report the first case of a delayed generalized skin reaction (urticaria and angioedema) occurring several hours after the first administration of denosumab. To our knowledge, no such adverse reaction after administration of this drug has previously been reported. We describe the first rapid desensitization protocol for denosumab. The cumulative therapeutic dose was reached in 2 h, and the protocol was successfully completed.

mAbs can cause infusion-related reactions but the exact etiology of these events remains unclear. They may arise via immunoglobulin (Ig) E- or non-IgE-dependent mechanisms [3]. Although an IgE-mediated mechanism was not confirmed by skin tests, our patient was empirically desensitized because the nature of the reactions indicated hypersensitivity. IgE- and non-IgE-sensitized patients may present with similar symptoms, indicating that mast cells and/or basophils are the cellular targets of these reactions. Nevertheless, in these patients, desensitization is a therapeutic procedure that aims to induce a temporary tolerance to the drug responsible for the hypersensitivity reaction. Rapid desensitization can be used for both IgE-mediated and non-IgE-hypersensitivity reactions [4].

The rates of hypersensitivity reactions that are clinically consistent with hypersensitivity to specific monoclonal antibodies have been reported to be 5–10 % for rituximab, 2–3 % for infliximab, and 0.6–5 % for trastuzumab [5]. Hypersensitivity reactions have also been reported for omalizumab, natalizumab, basiliximab, abciximab, adalimumab and cetuximab. In fact, desensitization protocols have been carried out with some of these biological agents, achieving therapeutic doses in patients who had presented anaphylactic or anaphylactoid reactions during treatment [6–8]. However, no guidelines for denosumab desensitization have been published to date.

A limitation of this study was the fact that the basophil activation test was not performed during the reaction. Nevertheless, the tryptase levels obtained indicated that there was no mast cell or IgE involvement.

In conclusion, we report the first case of generalized urticarial reaction and facial angioedema after therapy with denosumab. A subcutaneous desensitization protocol was successfully completed in this patient. The drug challenge results and the repeated high tolerance of our desensitization method without glucocorticoid premedication confirmed the etiology of reaction and the efficacy of the protocol, independently of the drug used to reduce adverse effects during the procedure. Rapid desensitization is a promising method for the delivery of monoclonal antibodies after hypersensitivity reactions, and should be considered in osteoporosis treatment when there are no acceptable therapeutic alternatives.
